# Pulsed electric field: A “green” extraction technology for biomolecular products from glycerol with fermentation of non-*Saccharomyces* yeasts

**DOI:** 10.3389/fbioe.2022.964174

**Published:** 2022-09-13

**Authors:** Evangelia A. Tsapou, George Ntourtoglou, Fotini Drosou, Panagiotis Tataridis, Stavros Lalas, Vassilis Dourtoglou

**Affiliations:** ^1^ Department of Wine, Vine and Beverage Sciences, School of Food Science, University of West Attica, Athens, Greece; ^2^ Department of Food Science and Nutrition, University of Thessaly, Karditsa, Greece

**Keywords:** pulsed electric field, non-saccharomyces yeasts, mannitol xylitol, propanediols, glycerol

## Abstract

Glycerol is the main organic by-product of the biodiesel industry and it can be a source of carbon for fermentations or a substrate for biotransformations. This work investigates a process that uses pulsed electric field (PEF) to enhance polyol and propanediols extraction from a glycerol/glucose fermentation broth. Three different commercial, non-*Saccharomyces* strains, *Torulaspora delbrueckii* Prelude (Hansen), *Torulaspora delbrueckii* Biodiva 291 (Lallemand) and *Metschnikowia pulcherrima* (Lallemand) were studied. The results revealed that PEF had a positive impact on the extraction of polyols ranging from 12 to 191%, independently of fermentation conditions. *Torulaspora delbrueckii* Biodiva 291 (Lallemand) was found to be more efficient at pH 7.1. An optimized chromatography-based method for the qualitative and quantitative determination of the formed products evaluated. The experiments were carried out either in flasks or in a bioreactor.

## Introduction

Pulsed electric field (PEF) is a technique that has been usedfor extractions due to its capability to cause disorganization of plant or microbial cells by disrupting membranes and releasing cell metabolites from the inner to outer part of the cell (Tsapou et al., 2020). The disruption of the cell membrane due to electroporation is caused by high intensity PEF and leads to complex phenomena ranging from cell restructuring to cell death (Delsart et al., 2012; Ntourtoglou et al., 2020). Electro-permeabilization of cells must be irreversible to cause inactivation of microorganisms. Electroporation was previously used as nonthermal treatment of liquid foods to inactivate microorganisms (Alvarez et al., 2003; Ntourtoglou et al., 2020; Tsapou et al., 2020).

Biofuels represent a category of fuels derived from biomass and include among others ethanol, biodiesel, green diesel and biogas. Biodiesel is considered to be a great substitute for conventional petro-diesel, which is a fast-depleting fossil fuel and for this reason is gaining worldwide popularity (Yang et al., 2012; Monteiro et al., 2018). During the transesterification process of the biodiesel production, approximately 10% of crude glycerol is formed as a by-product. Utilizing the glycerol waste is of outmost importance not only due to its potential to be used for the production of value added products but also due to the high cost and environmental impact that is associated with its disposal. It is known that a general ratio between biodiesel production and the amount of generated residual glycerol indicates that for every 10 parts of biodiesel, one part glycerol is produced (Mitrea et al., 2017). It is very important to convert crude glycerol into valuable byproducts to control and improve the economic sustainability of biodiesel production. Crude glycerol usually contains various impurities, such as water, methanol, soap, fatty acids, and fatty acid methyl esters. (Luo et al., 2016). Traditionally, crude glycerol was refined to pure glycerol, a useful raw material for industries, such as foods and beverages, pharmaceuticals, cosmetics, tobacco, and textiles (Luo et al., 2016; Samudrala, 2019). Different pathways, such as those involved in oxidation, acetalization, esterification, dehydration, and others used in the refining process yielding high added value products, can be obtained from crude glycerol (Aroua and Cognet, 2020).

Recent studies have focused on the use of crude glycerol as carbon source in microbial fermentations (Sadhukhan et al., 2016; Wischral et al., 2016; Mitrea et al., 2017). Many yeast, bacterial, and fungal strains are capable of growing on glycerol used as carbon source because this substrate can be both oxidatively and reductively metabolized through dehydrogenases or dehydratases (Mitrea et al., 2017). Some of the metabolic compounds that can be obtained via microbial fermentation of glycerol are acetic, lactic, propionic, citric, succinic, and oxalic acids, butanol, propanediols (1,3- and 1,2-propanediol), polyols (mannitol, xylitol, arabitol), ethanol, dihydroxyacetone, single-cell oil, biomass, and polyunsaturated fatty acids among others (Luo et al., 2016; Mitrea et al., 2017). Some of the microorganisms that have already been studied in aerobic and anaerobic fermentations with crude and pure glycerol as substrate are *Clostridium beijerinckii*, *Escherichia coli, Lactobacillus rhamnosus, Enterococcus faecalis Yarrowia lipolytica,* and *Lactobacillus brevis* (Murakami et al., 2016; Sadhukhan et al., 2016; Vivek et al., 2016; Wischral et al., 2016; Prabhu et al., 2020).

In the wine and beer industry selected non-*Saccharomyces* yeasts were observed to produce glycerol in addition to ethanol and volatiles during alcoholic fermentation (Drosou et al., 2022). However, most of works focused on ethanol production and sugar consumption and some of them on biogenesis of volatiles. Glycerol and polyols were analyzed either using enzyme assays or HPLC.

To increase the amount of the final products an electro-technique (PEF) was used in the post-fermentation extraction. High intensity of the fields induced by PEF have the effect of cell membrane disruption. This disturbance of the architectural structure of the membrane leading to a cell lysis for example or the fusion of protoplasts (Delsart et al., 2012; Tsapou et al., 2020). This phenomenon has resulted in pore formation in cell membranes and intercellular metabolites diffuse into the extracellular medium, due to a critical value of transmembrane potential, around 0.8–1 V (Zimmermann, 1986).

The aim of this work is the application of PEF in biotransformations by improving the extraction of certain bioactive compounds. Another aim is to investigate whether the results are associated with the efficiency of the fermentation process which is strongly influenced by various factors, including microbial characteristics (strain-microrganism, medium type and composition), as well as process parameters (e.g. power, frequency, electric field strength), treatment time, pH and temperature. Analytical techniques are also investigated. To conclude, studies under different operating conditions were made to verify the potential impact of PEF on the extraction of bioactive metabolites with the aim of optimizing the process to provide the optimum results for each application.

## Materials and methods

### Chemicals

Pyridine (Pyr), Acetic anhydride (AcOAc), diethyl ether (95%), anhydrous sodium sulfate, toluene, and hydrochloric acid (HCL), were purchased from Chem Lab (Athens, Greece). All reagents were of analytical quality. All the chemicals used for the preparation of the medium, were purchased from Sigma Aldrich (St. Louis, MO, United States).

### Fermentation

•**Yeast strains**

Three non-*Saccharomyces* yeast strains were used for the fermentation: 1. *Torulaspora delbrueckii* Prelude (Hansen) (Prelude), 2. *Torulaspora delbrueckii* Biodiva 291 (Lallemand) (Biodiva), and 3. *Metschnikowia pulcherrima* (Lallemand) (Mets).

•**Medium A**

For the preparation of the synthetic medium for the main cultures, the following ingredients were dissolved in 1 L of deionized water: 1 gl⁻^1^ of KH_2_PO_4,_ 1 gl⁻^1^ of K_2_HPO_4,_ 2 gl⁻^1^ of (NH_4_)_2_SO_4_, 0.2 gl⁻^1^ of MgSO_4_.7H_2_O, 0.2 gl⁻^1^ of ZnSO_4_.5H_2_O and yeast extract 1 gl⁻^1^. The medium was prepared the day of the fermentation 80 ml of the abovementioned medium was prepared and transferred to 150 ml flasks which were autoclaved at 121°C for 15 min. The cultures were initially inoculated with 1 ml of a 100 h pre-culture of the required strain (the concentration of the cells was calculated and adjusted at approximately 10 *10⁶ cells/ml). Cell counting was performed by microscopy, using a CX60 microscope (Olympus Corporation, Center Valley, United States) and a Thomas type hemocytometer. Viability was evaluated by the methylene blue method, according to Lange et al. (1993) and Drosou et al. (2021). Optical density (O.D.) for Biodiva and Prelude, were measured every day from zero time until the eighth day of the fermentation at 600 nm with a UV-Visible spectrophotometer (UV-1700 PharmaSpec, Shimadzu).

### Culture conditions for flask experiments

Fermentations were performed in 150 ml fermentation flasks which contained 80 ml of synthetic medium **A** supplemented with 62.64 gl⁻^1^ pure glycerol and 20 gl⁻^1^ glucose. Three different fermentation flasks were used each one containing one of the following microorganisms. A) *Torulaspora delbrueckii* (Prelude), B) *Torulaspora delbrueckii* Biodiva 291 (Biodiva) and C) *Metschnikowia pulcherrima* (Mets). The fermentations were performed under constant stirring (750 rpm) and at 25°C. From the second day till the fifth day 1 ml of pure glycerol was further added in each flask every 24 h. All fermentations were performed in duplicate. The weight of each fermentation flask was recorded daily; and the weight loss noted corresponded to CO₂ formation during the fermentation process. Samples were taken every 4 days to determine polyols. Polyols were analyzed at the end of fermentation (day 12 in all cases). pH settings was between 4.3 and 7.1.

### Culture conditions for fermentor experiments

For the needs of these experiments, a custom-made bioreactor was constructed. The bioreactor is comprised of three main parts.

**Part 1**: The first part was the main container of the bioreactor with a total volume of 2 L.

**FIGURE 1:**
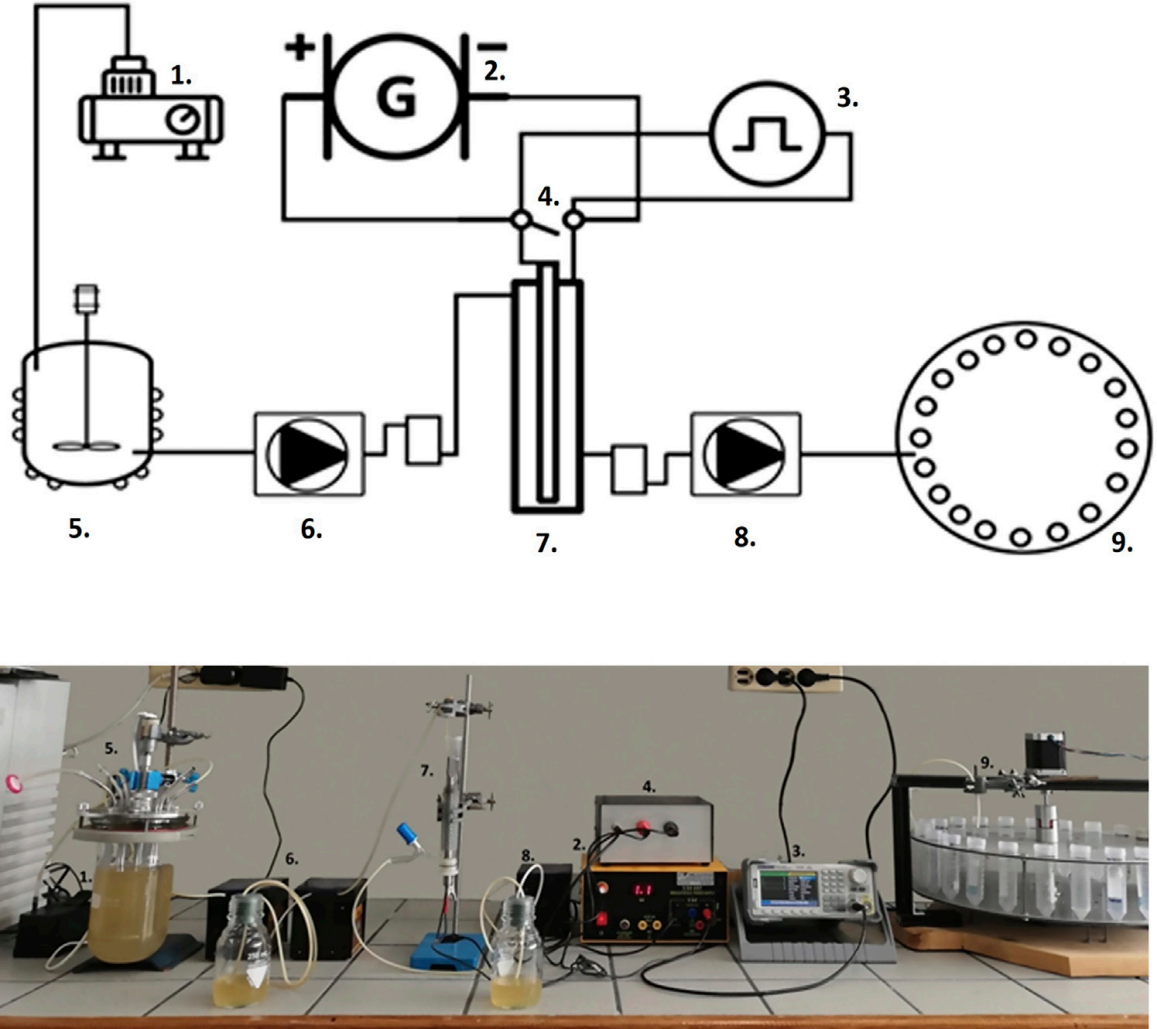
Process unit.

**Part 2:** On top of the main container mounted the lid of the bioreactor its outputs and inputs (Figure 1), which is airtight when closed. The lid consists of six ports, three on each side. All ports could be opened or closed at any given time.

• The two middle ports (3, 4) are connected and separated from the rest of the system by a stainless-steel pipe.
• Within the liquid inlet, another stainless steel pipe is installed, as well as a separate sieve assisting the separation in the sift of solid particles.
• To avoid the exposure of the thermometer to the reaction media, the thermometer placed inside a stainless steel tube, containing a thermal conductive media (glycerol, water, etc.)

Part 3: the third part was the stirrer also made out of stainless-steel (as exhibited on Figure 1) which was positioned inside the main hole. The stirrer had two Rushton Type 5 impeller tubes, one on each side, with a 5-cm diameter each. The motor can reach a maximum rotation speed of 200 rpm.

The total assembly in Figure 1 consisted of the bioreactor, an air pump, the PEF equipment with the treatment chamber as described by Ntourtoglou et al. (2020), one collector for the samples, and two peristaltic pumps that could push the fluid from the bioreactor to the PEF treatment chamber and consequently from the chamber to the sample collector.

**Table:** 

Nominal Volume, V (L)	2
Working volume, VL (L)	1.5
Impeller speed, TS (m/s)	0.5
Agitation speed, N (rmp)	60.00
Number of impeller	1
Impeller type	Rushton
Medium height, MH (cm)	10.5
Impeller diameter, DI (cm)	8
Reactor diameter, DT (cm)	20
Reactor hight, HT (cm)	10
MH/DT	2.12
DI/DT	0.30
Inoculum volume, Vx (0.1%)	0.001

In order to evaluate the bioreactor system, 1 L of the synthetic medium A enriched with 100 glL⁻^1^ glucose was prepared. The *Torulaspora* Biodiva was initially pre cultured in the same medium and then 1 ml of this preculture was used to inoculate the final medium in the bioreactor (concentration of the inoculum approximately at 10 *10^6^ cells/mL). The inoculum was added aseptically to the bioreactor. Pure glycerol was added at a rate of 5 ml/24 h from the third day onwards (Bioreactor Fermentation). The pH adjusted either at 4.3 or at 7.1 in two series of distinctive experiments.

### PEF equipment

The PEF equipment consisted of a high voltage generator (eisco, eshrrr 1,338 model), an IGBT control that is used as a high frequency switch and a pulse generator (siglent, SDG 1032X model) that controls the IGBT. The maximum voltage for the IGBT is 1100 V so the experiments took place with 1000 V for safety reasons. A square pulse with pick time 1 ms was created with a frequency of 1 Hz. The total treatment time was 30 min. The treatment chamber was a glass tube with 10 cm height and a diameter of 3 cm. Inside the tube, peripherally, there was perforated laminate stainless steel that took the shape of the exterior cylinder and in the middle there was a stainless steel cylinder with a diameter of 1 cm. The distance between the stainless steel cylinder and the perforated laminate stainless steel was 1 cm. The positive charge was in contact with the eccentric cylinder while the negative was joined with the peripherally perforated laminate stainless steel cylinder.

### Bioreactor combined with PEF extraction

For the fermentation and PEF extraction, the synthetic medium was enriched with 62.64 gl⁻^1^ pure glycerol and 20 gl⁻^1^ glucose. Pure glycerol was added at a rate of 1 ml/24 h for 4 days. Two bioreactors with 1 L medium and cells, as working volume, were cultured batch-wise for each pH value, one as the blank and the other consisted of a PEF treatment performed before the extraction of fermentation products.

### Sample preparation for GC/MS

20 ml of each sample was centrifuged twice at 18.33 m/s for 10 min to separate the phases (Hermle Z200A, Milan, Italy). Five milliliters of the supernatants were condensed in a flash evaporator (IKA RV 10) until a final concentration of 100 mg. Then the samples were mixed with Pyr and AcOAc at concentrations of 1,000 μl each, and left for 24 h in 63°C. After that, samples were extracted twice with 20 ml of diethyl ether and washed with 2 ml of 5% HCL and 1 ml of deionized water in a separation funnel. The organic layer was washed twice with 5 ml of distilled water dried over anhydrous sodium sulfate and filtered. Two milliliters of toluene were added twice to remove traces of Pyr and condensed until a concentration of ≈150 mg was achieved. Finally, 100 μl of dichloromethane was added to the samples from which 1.0 µL was used for GC/MS analysis. The results of all batches were presented in gL^-1^ and calculated by a standard triacetin curve.

### Gas chromatography/mass spectrometry analysis

The apparatus was an Agilent 6,890 series GC System (Agilent Technologies, Santa Clara, United States), equipped with 5975C VLMSD and an Agilent MG-HT-1 fused silica capillary column that was 30 m × 0.32 mm interior diameter (i.d.) × 0.25 μm film thickness. Moreover, samples were injected in a split ratio of 50:1. The injector temperature was set at 180°C, the carrier gas was helium at a flow rate of 1 ml/min, and the oven temperature was set initially at 50°C and was increased to 200°C at a rate of 10°C/min and maintained for 5 min. The temperature of the transfer line was set at 280°C. The mass spectrometer was operated in the ionization mode (EI) at an ionization voltage of 70 eV over masses ranging from 40 to 550 amu and a manifold temperature of 270°C. Data were recorded with Turbomass 5.0 ChemStation software (Agilent).

### Statistical analysis

Standard deviations were carried out with Excel 2013 (Microsoft, Redmond,WA, United States).

## Results and discussion

One of the objectives of this study was to optimize chromatography-based methods for polyol identification and quantification. After derivatization, using acetic acid anhydride, to synthetize polyols esters and Gas Chromatography coupled with Mass Spectrophotometry (GC-MS) seemed to be a powerful tool for polyol analyses in fermentation samples with or without residual sugars.

To evaluate the PEF technique as a downstream process in the extraction of products, more accurate product identification and analysis was necessary. From one point of view Gas chromatography/mass spectrometry (GC/MS) is an analytical technique, which gives accurate identification of the results. The conversion of products into their acetate esters provided an opportunity to be analyzed in more detail using with GC/MS (Table 1). From another point of view polyol esters are easily dissolved in organic solvents, thus, the extraction method used for the acetate esters combined with the PEF technique as a rapid “green” extraction technology was evaluated through the steps of microbial fermentation. Three strains of non-Saccharomyces yeasts were used to for the conversion of pure glycerol in biodiesel production.

**TABLE 1:** Results from the Blank and the fermentation in which a PEF treatment was held before the extraction. The results presented in gL⁻^1^.

	pH = 4.3		Difference	pH = 7.1		Difference
Compound	Blank	PEF		Blank	PEF	
1,2-Propanediol, diacetate	0.04	0.11	+175%	0.11	0.32	+191%
1,3- Propanediol, diacetate	0.72	1.40	+94%	0.68	1.30	+91%
1,2,3,4,5-Penta-O-acetyl-D-xylitol	0.20	0.56	+180%	1.20	1.90	+58%
D-Mannitol,hexaacetate	0.17	0.19	+12%	0.69	0.93	+35%
Triacetin	3.89	6.21	+60%	5.11	6.32	+24%

### Fermentation in flasks for optimal pH determination

**FIGURE 2:**
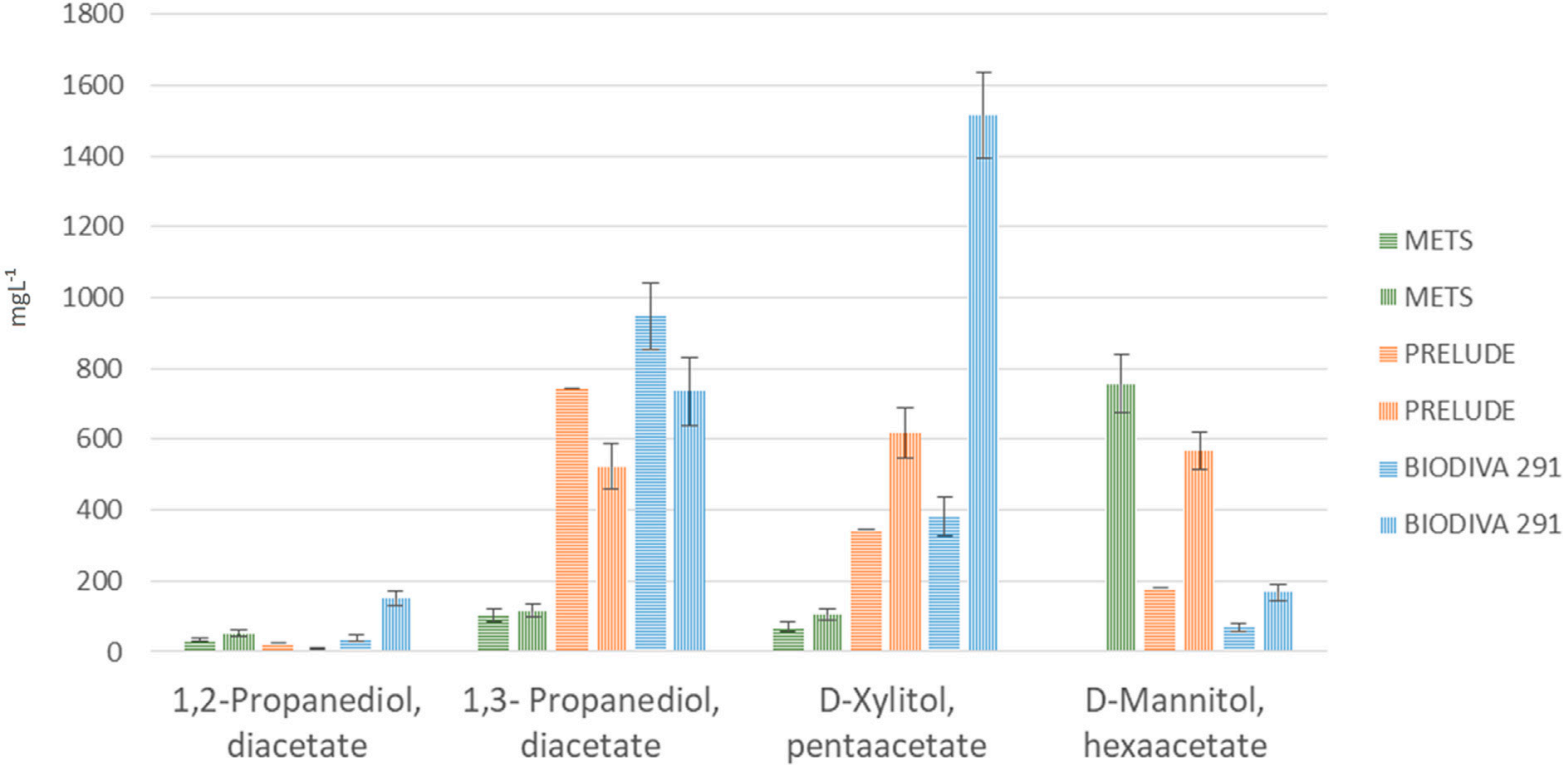
Results of the first series of flask fermentation (evaluation of yeast strains). Means of compounds in mgL⁻^1^ and standard deviations (n = 2).

From the first batch of fermentations, it was observed that all three strains were capable of producing propanediols and polyols during fermentation with glycerol (Figure 2). Concerning the efficacy of the fermentation at different pH levels, higher results for all strains were obtained in neutral pH. Mannitol and Xylitol are the polyols produced directly from glucose via its isomerization to fructose and they need the NADH/NADH₂ cofactor. As a result, a short aeration period is needed at the beginning of fermentation to avoid the displacement of NADH/NADH₂ equilibrium. In a previous work of Mbuyane et al. (2018), three strains of *Torulaspora delbrueckii* were used to produce polyols in a synthetic grape juice-like medium containing 120 gl⁻^1^ sugars. The results demonstrated that all strains were capable of producing polyols, such as d-arabitol, d-mannitol, d-xylitol, d-sorbitol, and ribitol. Previously Onishi and Suzuki (1968) stated that high concentrations of nitrogen sources and KH_2_PO_4_ in the medium remarkably decreased mannitol yield in spite of good utilization of the substrate using *Torulaspora. Mannitojuciens*. The produced mannitol was in yield of 31% consumed at optimal condition. They also stated that using washed yeast cells for the fermentation process gave much higher mannitol yield**.** Except for the carbon source, oxygen availability also studied during polyols production yield. Khan et al. (2009) investigated the production of mannitol from glycerol by resting cells of *Candida magnolia* and showed that oxygen availability influences positively the conversion, while potassium phosphate and excessive quantity of resting cells has a negative result.

Lee et al. (2003), mentioned that *Candida magnoliae* produced mannitol using a mixture of fructose and glucose, as carbon source, with a yield of 83%. They specifically achieved a production of 213 gL^-1^ mannitol in the fed-batch fermentation of *C. magnoliae* using a glucose:fructose mixture at a ratio of 1:20. *C. magnoliae* is also reported to produce glycerol (Sahoo and Agarwal, 2002), erythritol (Koh et al., 2003) and xylitol (Tada et al., 2004), using different substrates and at different fermentation conditions.

In the present study, xylitol was produced from all examined strains under both acidic and neutral conditions, while mannitol was detected only at neutral pH for Mets and Prelude fermentation and at both pH for Biodiva.

**FIGURE 3:**
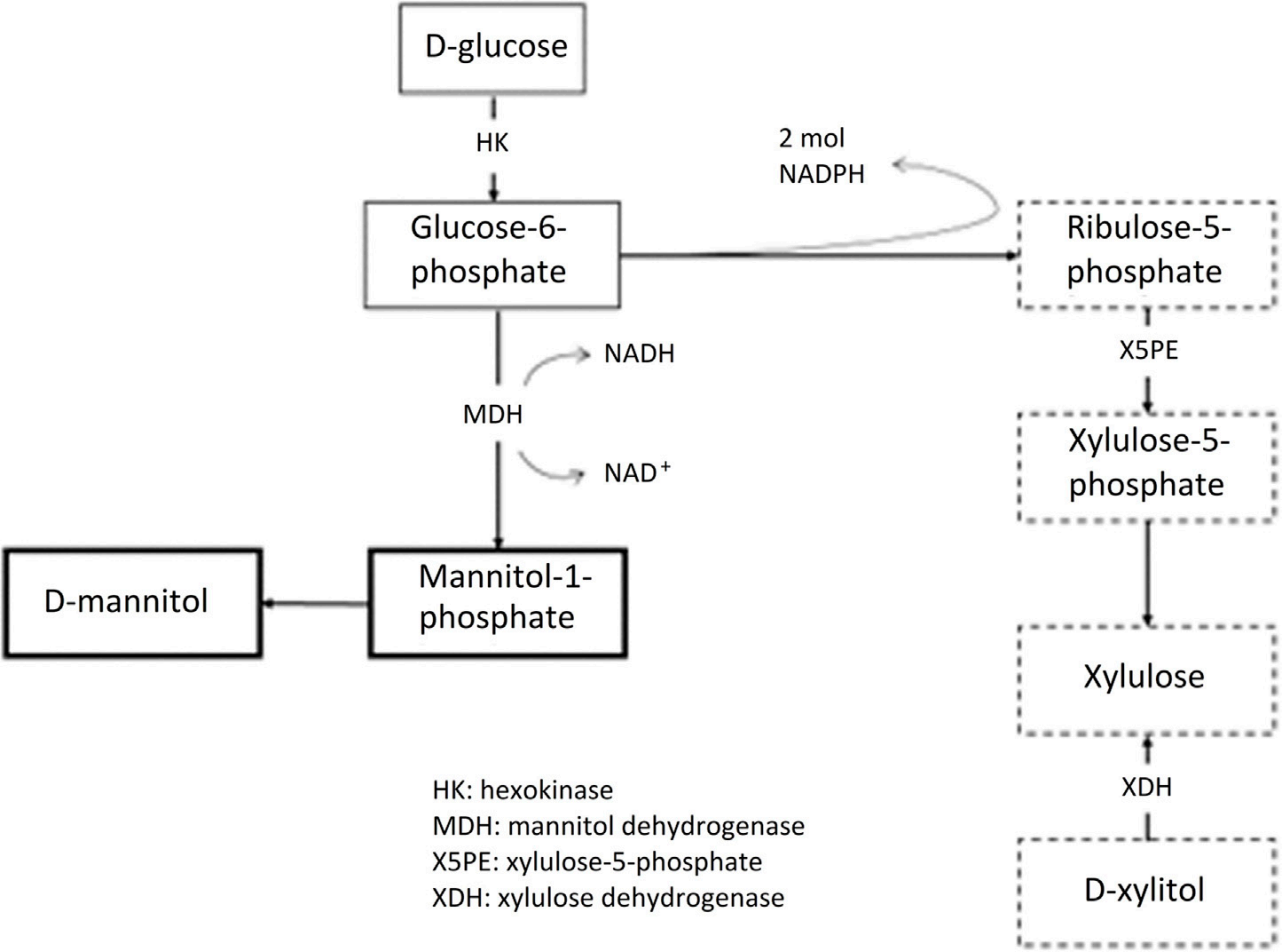
The pentose phosphate pathway and other metabolic routes for polyol production with glucose carbon source.

As the carbon source is not just glycerol but also a mixture of glycerol and glucose, it is normal to find all polyols present either from the glucose-fructose pathway (Figure 3) or from glycerol (propanediols). In addition, the influence of pH cannot be predicted and is derived from each experiment with different osmophilic yeasts.

**FIGURE 4:**
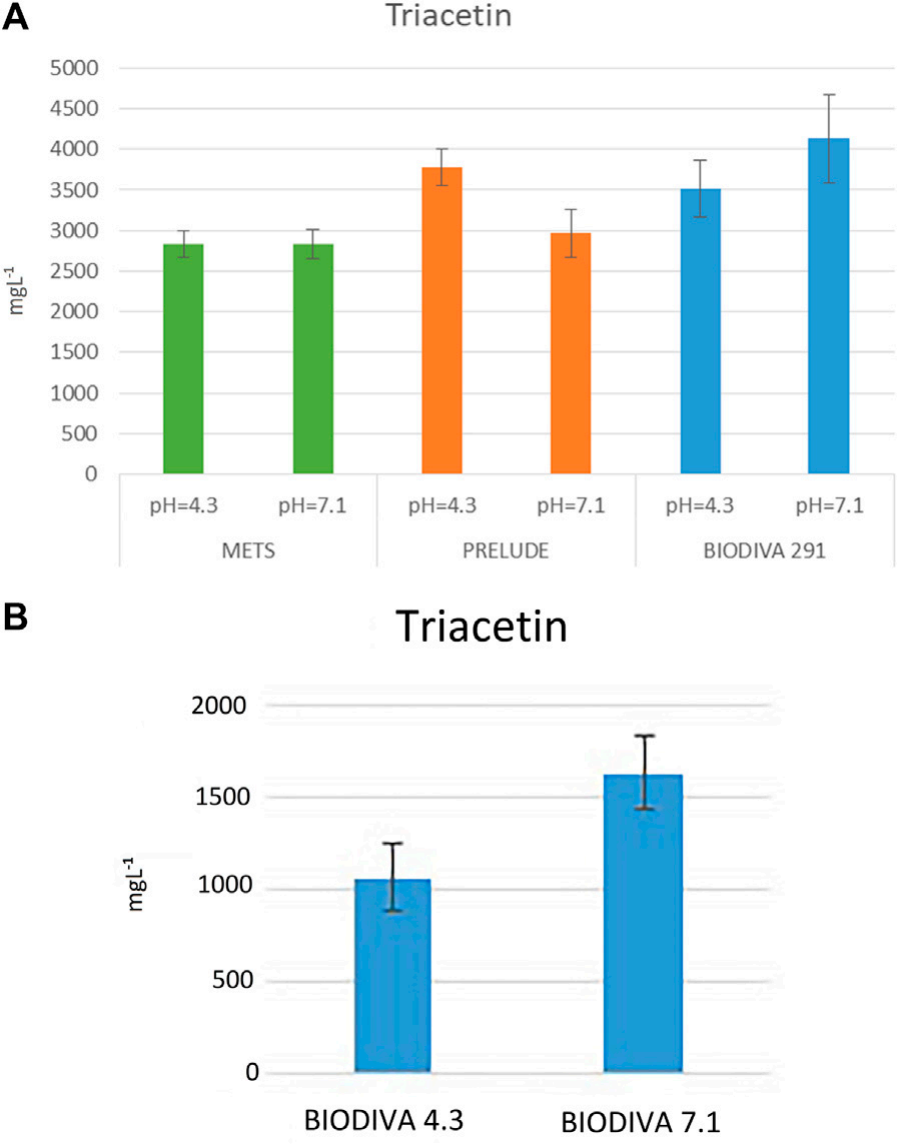
Evaluation of concumption of glycerin. Results of triacetin of **(A)** flask fermentation and **(B)** bioreactor fermentation, at pH 4.3 and 7.1. Means of triacetin in mgl⁻^1^ and standard deviations (n = 2).

The initial concentration of glycerol for all fermentations for this series of experiments was 62.64 gl⁻^1^. Comparing the concentrations of triacetin at the end of fermentation (Figure 4A), showed that the three strains were capable of consuming it as an energy source. Mets had the highest consumption while showing the lowest concentrations of 1,3-propanediol, mannitol, and xylitol. In Biodiva fermentation, higher amounts of the same products were analyzed, while having the lowest consumption efficiency of triacetin at 93% compared with the two other species (Figure 2). In Prelude fermentation, equal amounts of both glucose and triacetin were detected, a fact that might indicate that this strain needs more than 1 week of fermentation to yield the corresponding results.

Mannitol and xylitol were quantified at higher concentrations in Biodiva samples. Specifically, for acidic pH, xylitol was found to be approximately five times higher in Prelude and Biodiva when compared with Mets. On the other hand, in a neutral pH, these two polyols were found at levels approximately fifteen times higher than in Biodiva and six times higher than in Prelude when compared with Mets. Concerning mannitol in an acidic pH, was found at a concentration of 0.18 gl⁻^1^ only after Biodiva fermentation. In a neutral pH, all strains were found to have mannitol, with the highest concentration (0.76 gl⁻^1^) in Biodiva samples (Figure 2).

Both propanediols, 1,2- and 1,3-propanediol, were analyzed from all three strains at both pH values. 1,3-Propanediol had a higher concentration than 1,2-propanediol in all fermentation products. In Mets fermentation, the concentrations did not show much difference according to pH. In contrast, for Prelude and Biodiva, higher amounts of 1,3-propanediol were found at neutral pH. The higher concentrations found in Biodiva were 0.95 and 0.74 gl⁻^1^ at pH values of 4.3 and 7.1, respectively, for 1,3-propanediol and for 1,2-propanediol quantified in 0.15 and 0.04 gl⁻^1^ for pH 4.3 and 7.1, respectively. In the eighth day of fermentations (end time point), dry weight was measured for Biodiva and Mets and it was found to be 400 and 328 mg respectively. According to the results, Biodiva modified the medium into valuable products in higher amounts during a 1-week fermentation period.

### Fermentation in bioreactor

**FIGURE 5:**
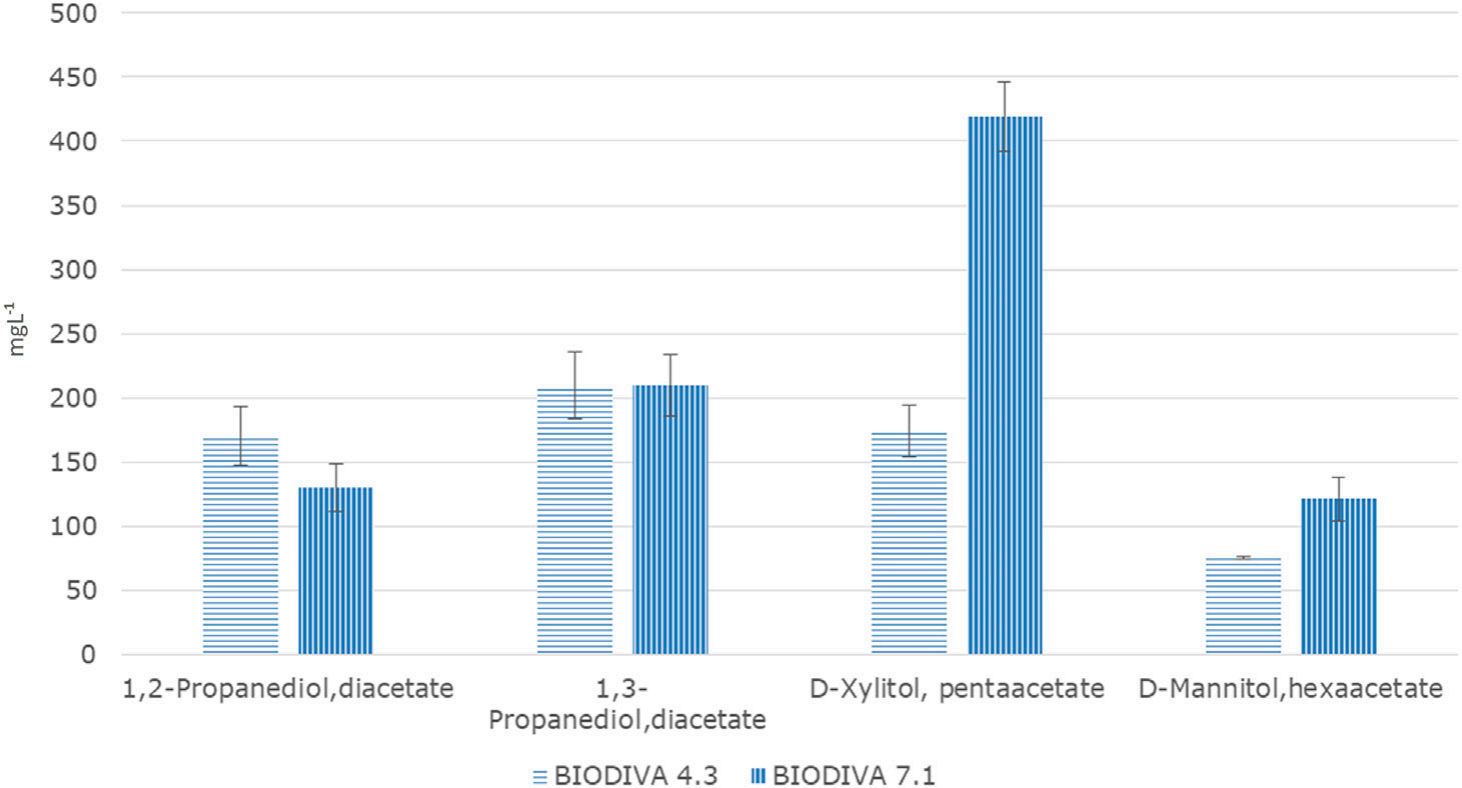
Results of bioreactor fermentation (evaluation of yeast strain in various pH). Means of compounds in mgL⁻^1^ and standard deviations (n = 2).

**FIGURE 6:**
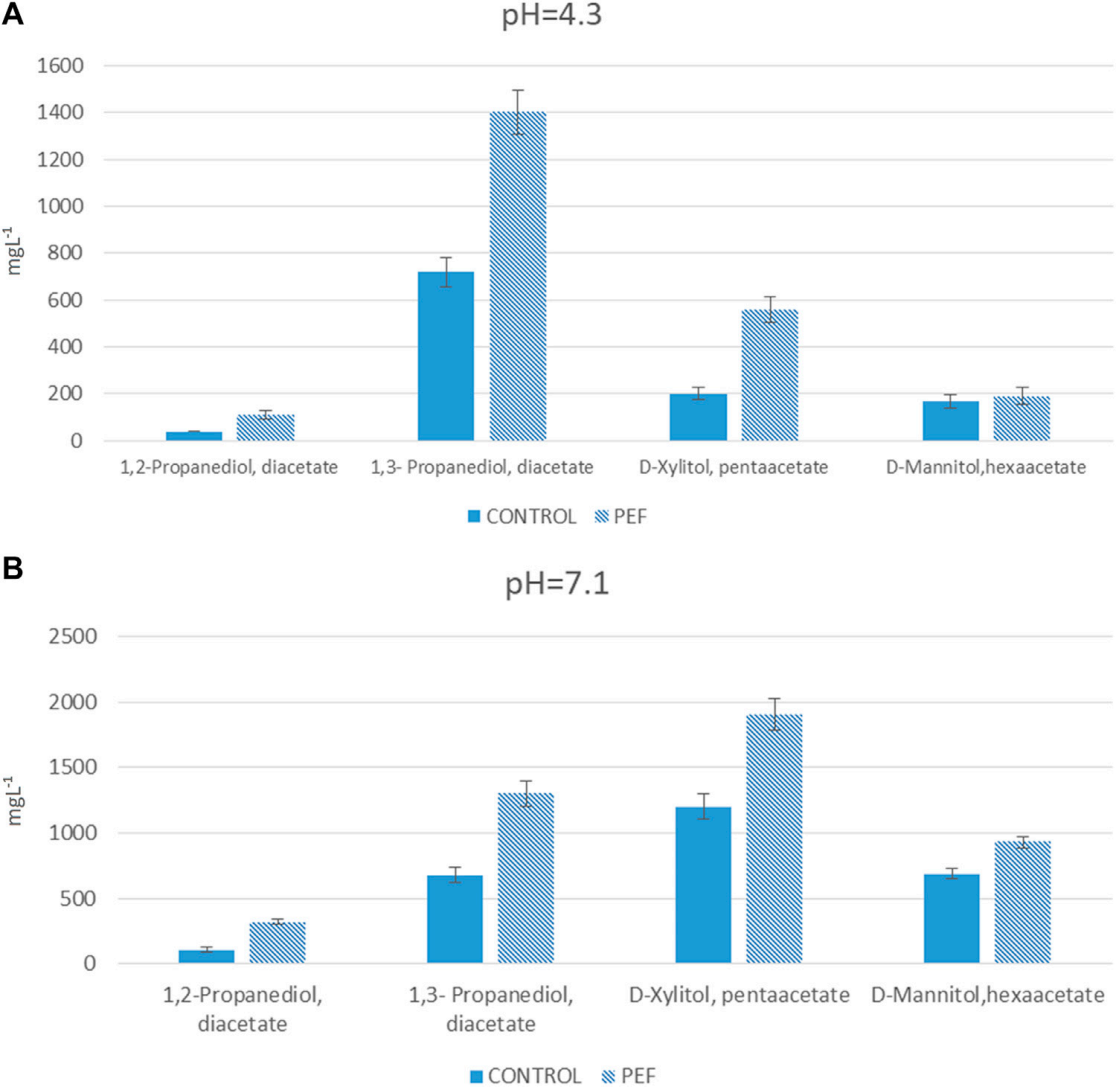
Results of bioreactor fermentation and PEF assisted extraction of polyols, at pH **(A)** 4.3 and **(B)** 7.1. Means of compounds in mgL⁻^1^ and standard deviations (n = 2).

In order to scale up the fermentation, validate the pH of medium composition, and the source of the carbon, bioreactor cultivations were carried out in a 2.00 L scale bench bioreactor with 1 L working volume. The process conditions were identical as in the shake flask studiy except for the aeration. The aeration rate was maintained at 2.0 L/min for the first 72 h and then stopped. Two separate batch fermentations were ran, with the Biodiva strain. One at pH 4.3 and using glucose/glycerol as the carbon sources and the other at pH 7.1 with the same carbon substrates. The time course profiles for both fermentations were similar to that of the shake flask cultivations. Glycose is the most preferred carbon source for Βiodiva and in the presence of glycerol the uptake of xylose was repressed as evident in Figure 2 and Figures 5, 6. NADP/H2 availability is in most cases a rate-limiting factor in the reduction of xylose to xylitol.

There are three ways that microbial cells produce polyols. The first is a direct hydroxyl elimination leading to purivate derivatives propane diols, the second is an isomerization of glycose to maltitol and other polyols and the last one is from ribulose 5-phosphate, as shown in Figure 2. In the work of Tavares et al. (1999) it is shown for the first time that xylitol production by the yeast *Debaryomyces hansenii* is not only a result of a redox balance usually occurred under poor aerobic conditions, but also that there are additional physiological mechanisms involved, mainly from phosphate limitation.

**FIGURE 7:**
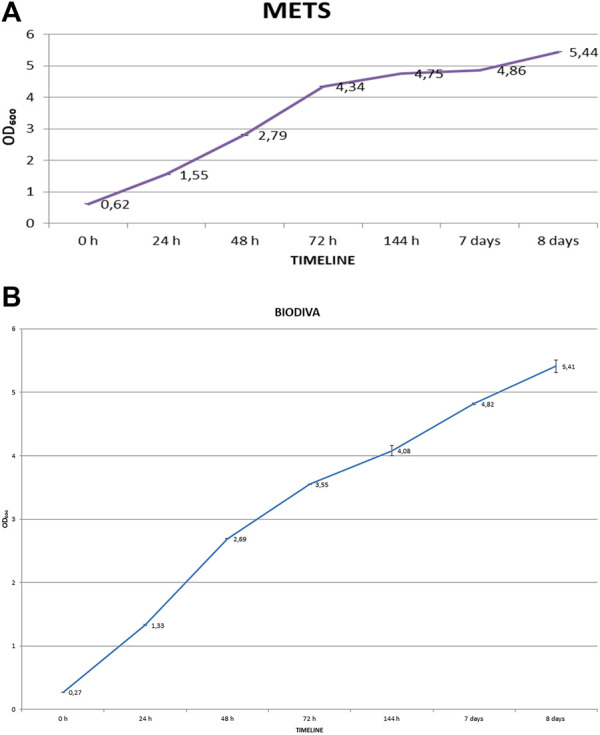
Time course profiles of **(A)** *Metschnikowia pulcherrima* and **(B)** *Torulaspora delbrueckii* on OD₆₀₀, for flask cultivation. Means and standard deviations (n = 2).

In the next series of experiments, the bioreactor was used for the Biodiva fermentation process in the glucose-glycerol medium to allow the first step for the scale up of the production polyols from laboratory bench to industrial levels (Figure 4). In this batch, the medium contained 100 gl⁻^1^ of glucose and 23.2 gl⁻^1^ of glycerol. From Figures 4, 7, it was concluded that the consumption efficiency of glycerol was 93% for neutral pH and 95% for the acidic pH. The concentration of triacetin in addition to the initial concentration of glycerol was lower when compared with the results from the shake flask studies, indicating that the consumption of glycerol at acidic pH was higher, but the amounts of polyols were lower when compared with neutral pH. Better results were obtained again at neutral pH.

Specifically, for propanediols, their concentrations were almost the same with no significant difference at either pH values. As a sum, their concentrations were lower with the flask fermentation (for Biodiva), 0.38 gl⁻^1^ and 0.39 gl⁻^1^ for acidic and neutral pH values, respectively, while in flask fermentation, the concentrations were 0.99 gl⁻^1^ and 0.89 gl⁻^1^, respectively. In polyol production, an increase in this batch was noted, which was lower for mannitol and higher for xylitol, and at neutral pH yielded better results for both polyols. As a sum of the concentration of these two polyols and after comparing flask and bioreactor fermentation, an increase in the results from bioreactor fermentations was found. Particularly, in acidic pH, the increase was 42.8%, while in neutral pH the increase was 20.4%.

### Application of PEF for increase the extractability of polyols

For the last experiment, a third batch was examined. At both neutral and acidic pH levels, the concentration of valuable compounds in the PEF samples were higher. Most of the metabolites are intracellular, such as polyols (Kasumi, 1995), and this is the main reason of the evaluation of PEF, to enhance polyol extraction from a glycerol/glucose fermentation broth. The most important difference was found between the blank and PEF samples with percentages ranging between 191 and 12%. Specifically, the percentage of mannitol in neutral pH was improved by 35% after the PEF treatment and in acidic pH by 12%. Finally, from the initial amount of glycerol that was added at the start of the experiment, an average of 91% was consumed with the highest value in acidic pH in the blank and lowest in neutral pH in the blank. The highest concentration of triacetin in both PEF samples was normal because the yeast cells produce glycerol to maintain a stable osmotic level. It is well known that during PEF treatment, the cell membrane expands and the products that are trapped inside the membrane escape into the environment (Angersbach et al., 2000).

## Conclusion

In conclusion, it was demonstrated that the use of PEF could increase the yield of the fermentation products of three non*-Saccharomyces* yeast strains using glycerol/glycose as a carbon source. The experiment was performed both at small volumes (flasks) and at bioreactors (as an up-scaling process). Between the three non-*Saccharomyces* yeasts, Biodiva 291 (Lallemand) (Biodiva) was the strain which seemed to be more efficient in the biotransformation process. According to our findings, the PEF-treated samples showed higher concentrations of compounds in both acidic and neutral environments. The PEF treatment was shown to enhance the concentration of compounds after fermentation from 12 to 180% in an acidic environment and from 24 to 191% in a neutral environment. Specifically xylitol has an increase of 180% in acidic pH and 1,2-propanediol has an increase of 175 and 191% in acidic and neutral pH respectively. From our results, we can reach the conclusion that the PEF treatment aids and enhances the isolation of the fermentation products. Therefore should be suggested that this technique has the potential to be used for industrial applications and should be further investigated.

## Data Availability

The original contributions presented in the study are included in the article/Supplementary Material, further inquiries can be directed to the corresponding author.
